# A Gene Catalogue of the Euchromatic Male-Specific Region of the Horse Y Chromosome: Comparison with Human and Other Mammals

**DOI:** 10.1371/journal.pone.0021374

**Published:** 2011-07-25

**Authors:** Nandina Paria, Terje Raudsepp, Alison J. Pearks Wilkerson, Patricia C. M. O'Brien, Malcom A. Ferguson-Smith, Charles C. Love, Carolyn Arnold, Peter Rakestraw, William J. Murphy, Bhanu P. Chowdhary

**Affiliations:** 1 Department of Veterinary Integrative Biosciences, Texas A&M University, College Station, Texas, United States of America; 2 Centre for Veterinary Science, University of Cambridge, Cambridge, United Kingdom; 3 Department of Large Animal Clinical Sciences, Texas A&M University, College Station, Texas, United States of America; Temasek Life Sciences Laboratory, Singapore

## Abstract

Studies of the Y chromosome in primates, rodents and carnivores provide compelling evidence that the male specific region of Y (MSY) contains functional genes, many of which have specialized roles in spermatogenesis and male-fertility. Little similarity, however, has been found between the gene content and sequence of MSY in different species. This hinders the discovery of species-specific male fertility genes and limits our understanding about MSY evolution in mammals. Here, a detailed MSY gene catalogue was developed for the horse – an odd-toed ungulate. Using direct cDNA selection from horse testis, and sequence analysis of Y-specific BAC clones, 37 horse MSY genes/transcripts were identified. The genes were mapped to the MSY BAC contig map, characterized for copy number, analyzed for transcriptional profiles by RT-PCR, examined for the presence of ORFs, and compared to other mammalian orthologs. We demonstrate that the horse MSY harbors 20 X-degenerate genes with known orthologs in other eutherian species. The remaining 17 genes are acquired or novel and have so far been identified only in the horse or donkey Y chromosomes. Notably, 3 transcripts were found in the heterochromatic part of the Y. We show that despite substantial differences between the sequence, gene content and organization of horse and other mammalian Y chromosomes, the functions of MSY genes are predominantly related to testis and spermatogenesis. Altogether, 10 multicopy genes with testis-specific expression were identified in the horse MSY, and considered likely candidate genes for stallion fertility. The findings establish an important foundation for the study of Y-linked genetic factors governing fertility in stallions, and improve our knowledge about the evolutionary processes that have shaped Y chromosomes in different mammalian lineages.

## Introduction

Mammalian Y chromosome stands out from the rest of the genome because it is male specific, constitutively haploid and exhibits unique structural and functional features [1]–[3]. Typically, it is one of the smallest chromosomes in the genome and harbors both the pseudoautosomal and the male-specific genes.

The male specific region of the Y (MSY) has been sequenced in the human (*Homo sapiens*, HSA) [1] and the chimpanzee (*Pan troglodytes*, PTR) [4] leading to systematic discovery and mapping of male-specific genes. Detailed functional characterization of Y chromosome genes has been carried out also in mouse (*Mus musculus*, MMU) [5]–[10]. Additionally, Y chromosome genes have been mapped and/or functionally analyzed in carnivores [11]–[12] and cattle [13]–[16], and to a limited extent in river buffalo [17], pig [18]–[19], sheep [20]–[21] and rabbit [22]. These studies clearly show that mammalian Y chromosomes carry a rich repertoire of functional genes, several of which might play a role in spermatogenesis and male fertility.

The human MSY euchromatin contains 27 gene families corresponding to 78 transcriptional units [1]. Another eight genes with open reading frames (ORFs) are located in a small euchromatic island in the HSAY pericentromeric heterochromatin [23]. Compared to humans, the PTRY has lost large fractions of MSY protein-coding genes in the course of evolution, and has retained only 18 gene families with 37 transcriptional units [4]. Gene catalogues for other mammalian Y chromosomes are less comprehensive: 53 genes or gene families have been identified in mouse (http://www.ncbi.nlm.nih.gov/mapview/), 19 in cat [11]–, over 13 in cattle [13]–[16] and at least 16 in gorilla [24]. The mouse Y chromosome is functionally well studied, and provides compelling evidence that Y-linked genes are involved in multiple processes during spermiogenesis, including sperm motility, and the development and function of the acrosome [5], . In cat (*Felis catus*, FCA), the MSY genes have been analyzed for expression profiles and possible involvement in male fertility [11]–[12]. The organization of FCAY resembles that of MMUY as single copy genes contain on the short arm, while highly amplified testis-specific gene families are distributed all over the long arm [12]. Considerable progress has been made in mapping and analyzing the cattle (*Bos taurus*, BTA) Y chromosome including the construction of basic RH [13] and cytogenetic maps [28], and identifying over 10 X-degenerate genes and three lineage-specific gene families, *viz.*, *ZNF280BY, ZNF280AY* and *PRAMEY* [15]-[16]. Among other domestic species, pig is probably the next to have a high resolution Y chromosome map and sequencing data. This is because Pig Genome Sequencing project is based on a whole genome BAC fingerprint contig including the Y chromosome [29].

Human and chimpanzee MSY sequences demonstrate that most of the Y chromosome genes fall into two sequence classes: ampliconic (multicopy gene families expressed predominantly or exclusively in testis) and X-degenerate (single copy ancestral homologues of X-linked genes, most of which are expressed ubiquitously) [1], [4]. However, there are pronounced differences between mammalian Y chromosomes studied so far, and MSY sequences can differ even between closely related species. For example, over 30% of the chimpanzee MSY sequence has no counterpart in the human MSY, and the PTRY has only two-thirds as many distinct genes as HSAY [4]. Comparisons between species show that a core set of X-degenerate genes are shared between mammals, while multicopy genes, which are typically Y-borne or acquired from other parts of the genome, are species- or lineage-specific [1], [4], [11], [30]. Furthermore, the majority of testis expressed, and potentially male fertility-related MSY genes are restricted to a species or a group of related species [10]–. The unusual features of the MSY call for the systematic discovery of Y-linked genes in a larger number of mammalian species, mainly to improve our knowledge about the evolutionary processes that have shaped the MSY gene content in different lineages, and to identify species-specific male fertility genes.

While stallion fertility is of prime importance to the equine industry, the current knowledge about Y-linked fertility factors in horses is limited, thus justifying the launch of systematic Y chromosome research in this species. Given that the domestic horse (*Equus caballus*, ECA) is a eutherian mammal that is evolutionarily distant from primates, rodents and carnivores (http://www.timetree.org/), the findings will bear also comparative value.

The first gene loci, *viz., TSPY* [31], *ZFY* [32] *AMELY* [33] and *SRY* [34] were assigned to the horse Y chromosome (ECAY) indirectly by PCR. Thereafter, *SRY*, *ZFY,* and *STS* were synteny mapped to ECAY by somatic cell hybrid analysis [35], and the location of *SRY* and *ZFY* was further refined using FISH [36]. Systematic discovery of ECAY genes, however, started in 2004 when the location and linear order of eight MSY genes was determined using a combination of radiation hybrid analysis, FISH, and BAC contig mapping [37]. This was followed by a detailed mapping of the pseudoautosomal region [38]. Altogether, these studies have identified 28 Y-linked genes in horses of which majority are pseudoautosomal [38], while only 9 are present in the MSY [37]. In the present study, we employed direct cDNA selection [39] from equine testis, followed by isolation and analysis of MSY specific cDNA clones to obtain a collection of male-specific genes in the horse. The genes were tentatively assigned to the ECAY contig map, and analyzed for various structural and functional features. The gene catalogue of equine MSY allowed us to make evolutionary inferences with other eutherian species, and to identify Y-linked candidate genes for stallion fertility.

## Results and Discussion

### Discovery of horse Y chromosome genes and transcripts

**Figure 1:**
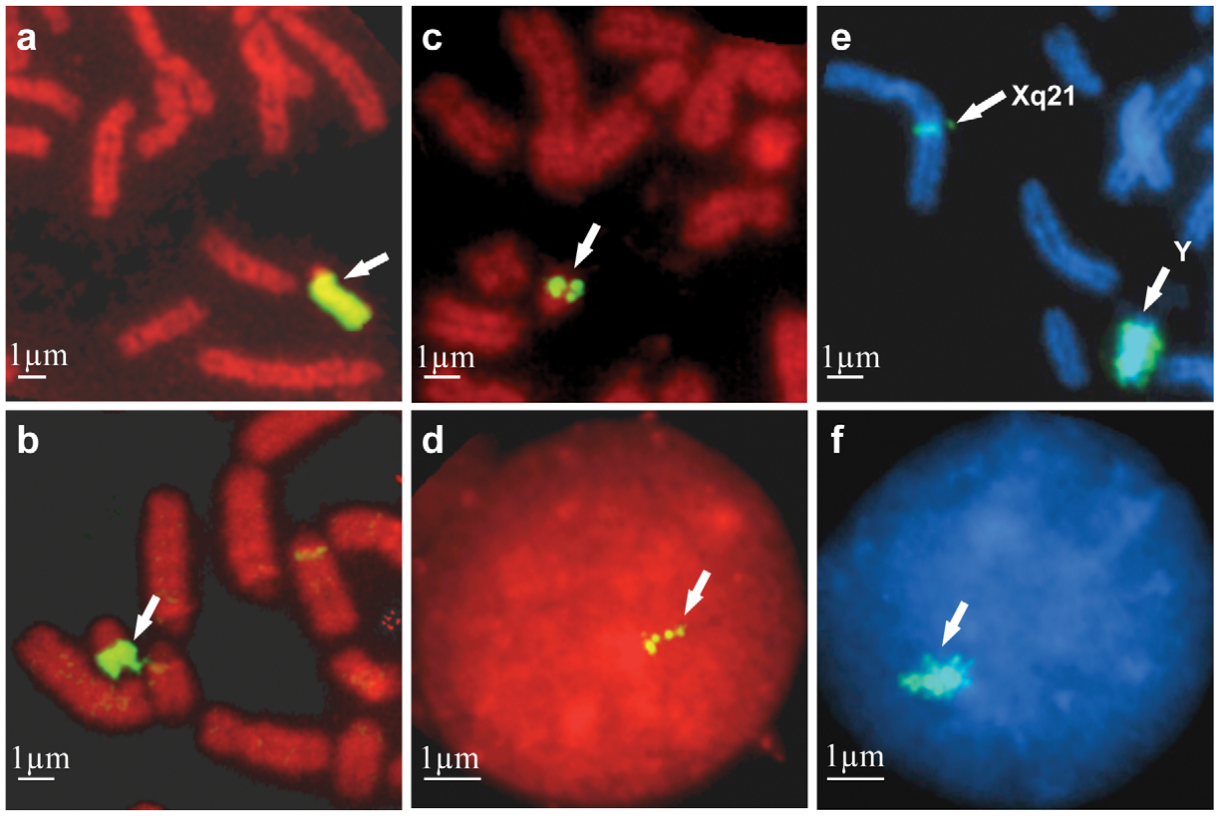
Fluorescence *in situ* hybridization (FISH) using ECAY probes. **a.** painting-like signal with flow-sorted Y probe on ECAY eu- and heterochromatin; **b.** painting-like signal with a probe comprising of 33 MSY BAC clones on ECAY euchromatin; **c.** and **d.** distinct signals with *UBE1Y* cDNA in metaphase and interphase Y chromosome; **e.** and **f.** painting-like signals with *ZNF33bY* cDNA on ECAY and ECAXq21 heterochromatin in metaphase and interphase. Scale bar, 1 µm.

We selected cDNAs from equine testis using horse Y chromosome specific composite probes as *selectors*, following the methodology previously described for human [40]–[41] and cat [11]–[12]. The purity of the *selectors* (the flow sorted ECAY and MSY BAC pools) was confirmed by FISH (Fig. 1a, b). After selection, a plasmid library enriched with ECAY cDNA sequences was constructed. We picked 2,400 clones for Sanger dideoxy sequencing, and obtained 1,678 quality sequences that were assembled into 180 contigs and 100 singletons. PCR analysis using contig/singleton-specific primers on male and female genomic DNA showed that 30 contigs and 74 singletons were male specific whereas 150 contigs and 26 singletons amplified both from male and female genomic DNA. Majority of the latter showed significant similarity to known autosomal genes, as well as to autosomal and X-chromosome genomic assemblies, and were removed from further analysis.

Fourty-four male-female specific cDNA sequences that did not show any significant similarity with known autosomal genes were analyzed by PCR on BAC clones that form the minimum tiling path of the equine pseudoautosomal region (PAR) [38]. No equine PAR transcripts were recovered. Given that a comparable cDNA selection procedure identified all 9 human PAR genes known at the time [40], the results in horse were unexpected. It is, though, possible that some genomic assemblies corresponding to the X chromosome as detected by BLAST analysis, contained PAR genes which remained unidentified. Therefore, the failure to recover PAR genes in this study is likely because of biased data analysis and not due to low efficacy of cDNA selection procedure.

We identified 321 (19%) cDNA sequences to be male specific by PCR amplification of male and female genomic DNAs using cDNA-specific primers. Among these we found significant (<1e-8) similarity with 13 mammalian MSY and 3 autosomal genes (Table S1). PCR primers for the latter, *i.e. EIF3CY, RPS3AY,* and *ZNF33bY,* amplified two distinct products from genomic DNA: a larger autosomal product, which was present in males and females, and a smaller male specific product (Fig. S1). DNA sequencing showed that the two bands differed by a small internal deletion in the Y-derived sequence, though it was not possible to design exclusively male-specific primers. Ten male-specific transcripts were considered novel and horse-specific because no sequence similarity was found in any other species studied so far. Following transcriptional analysis (see below), these sequences were labeled as *ETSTY1*-*6* (Equine Testis-Specific Transcript on Y) and *ETY1*-*4* (Equine Transcript on Y) indicating whether they had testis-specific or broader tissue expression profiles (Table 1). Finally, we found cDNA sequences with a weak similarity (>1e-8) to mitochondrial *MT-ND1*, autosomal *RFX5* and X-degenerate *EIF1AY* genes. Thus, these annotations remained tentative.

**Table 1:** Summary information about the horse MSY genes and transcripts.

Gene Symbol	Copy number		Evolutionaryorigin	Expressionprofile	No. of cDNAs	ORF	References
	BAC	cDNA					
*AMELY*	SC	n/a	X degenerate	None	0	Yes	[33]
*ATP6V0CY*	SC	n/a	Acquired	U	0	No	[42]
*CUL4BY*	MC	n/a	X degenerate	I: *Td, H, K, S*	1	Yes	this study
*CYorf15*	SC	n/a	X-degenerate	U	2	Yes	this study
*DDX3Y*	SC	n/a	X degenerate	U	2	Yes	[37]
*EIF1AY*	SC	n/a	X-degenerate	U	2	No	this study
*EIF2s3Y*	SC	n/a	X degenerate	U	0	No	this study
*EIF3CY*	SC	n/a	Acquired	U	85	Yes	this study
*ETSTY1*	MC	MC	Y borne, novel	T	2	No	this study
*ETSTY2*	MC	MC	Y borne, novel	T	7	Yes	this study
*ETSTY3*	MC	MC	Y borne, novel	T	12	Yes	this study
*ETSTY4*	MC	MC	Y borne, novel	T	9	Yes	this study
*ETSTY5*	MC	MC	Y borne, novel	T	3	Yes	this study
*ETSTY6*	HC	HC	Y borne, novel	T	2	Yes	this study
*ETY1*	MC	MC	Y borne, novel	I: *Td, B, H, K, Li, Lu, S*	1	Yes	this study
*ETY2*	SC	-	Y borne, novel	U	1	No	this study
*E(T)Y3*	HC	HC	Y borne, novel	None	1	No	this study
*ETY4*	MC	MC	Y borne, novel	U	1	Yes	this study
*KAL1Y*	SC	-	X degenerate	None	0	No	this study
*KDM5D*	SC	-	X degenerate	U	3	Yes	[84]
*MAP3K7IP3Y*	SC	-	X degenerate	U	0	No	this study
*MT-ND1Y*	SC	-	Acquired	U	1	No	this study
*NLGN4Y*	SC	-	X degenerate	I: *B, SV, T*	1	No	this study
*RBMY*	MC	MC	X degenerate	T	1	No	this study
*RFX5Y*	SC	-	Acquired	I: *K, Li, S, T*	1	No	this study
*RPS3AY*	SC	-	Acquired	U	1	Yes	this study
*SRY*	MC	SC	X degenerate	I: *Td, K, SV*	43	Yes	[34]
*STS-Y*	SC	-	X degenerate	n/a	0	n/a	[35]
*TBL1Y*	SC	-	X degenerate	n/a	0	n/a	this study
*TMSB4Y*	SC	-	X degenerate	U	1	No	this study
*TSPY*	MC	MC	X degenerate	T	12	Yes	[31]
*UBE1Y*	MC	MC	X degenerate	T	54	Yes	this study
*USP9Y*	SC	-	X degenerate	U	3	Yes	[37]
*UTY*	SC	-	X degenerate	U	0	No	[37]
*YIR2*	MC	MC	Y borne	I: *Td, H, K, Li, Lu, S*	1	No	this study
*ZFY*	SC	-	X degenerate	U	3	No	[32]
*ZNF33bY*	HC	HC	Acquired	T	64	No	this study

**Copy number:** SC – single copy; MC- multicopy; HC – heterochromatic; **Expression:** T-testis; Td – testis predominant; I – intermediate; U- ubiquitous; n/a – no data. **Tissues:** B-brain; K-kidney; H-heart; SM-sceletal muscle; Li-liver; Lu-lung; S-spleen; SV–seminal vesicle.

Although the search for expressed sequences by direct selection was systematic, some previously mapped MSY genes [37] escaped detection. Thus, 8 genes (Table S1) were identified exclusively from MSY BAC clones by PCR with gene specific primers [37], or by analyzing BAC end or whole BAC [42] sequences. Because cDNA selection retrieves expressed sequences from a particular tissue, sequences which are not transcribed in this tissue, or have a low relative transcription rate, remain undetected. Indeed, *AMELY* which is known to be expressed only in developing tooth buds [43], was not recovered from equine testis cDNA. Further, it is possible that transcription levels of *ATP6V0CY, EIF2s3Y, MAP3K7IP3Y, UTY,* and *TBL1Y* in horse testis were too low for detection, while *STS-Y* and *KAL1Y*, like in human [1], might be Y-linked pseudogenes in the horse. Altogether, we identified a total of 37 MSY genes and transcripts in the horse, 29 of them were isolated by direct cDNA selection while 8 genes were discovered by BAC analysis.

### The horse MSY gene map

**Figure 2:**
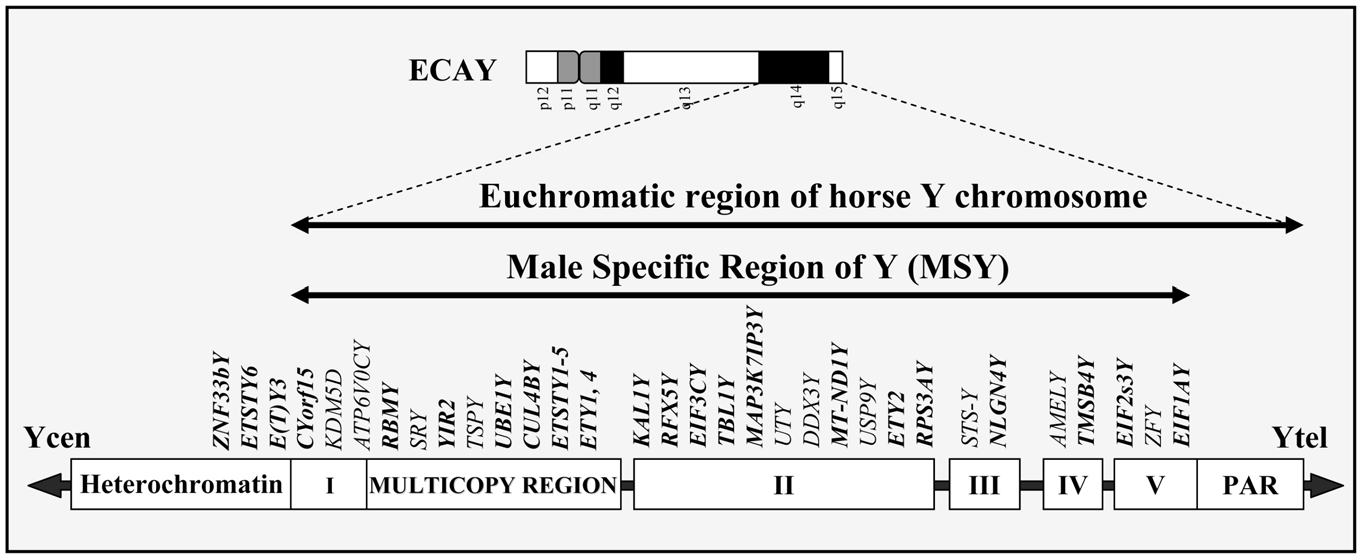
A gene map of the horse male-specific region on Y (MSY). A G-banded ideogram (ISCNH 1997) of the horse Y chromosome (ECAY) is shown at the top. Positive G-bands are black, negative G-bands are white and the centromere is shown in grey color. Horizontal lines below the ideogram demarcate the span of the euchromatic and male specific regions of Y. A schematic of ECAY contig map [83] is at the bottom. White blocks with Roman numerals demarcate the currently available five BAC contigs. Heterochromatic region is shown at the far left and the pseudoautosomal region at the far right. The approximate locations of the 37 MSY genes are shown at the top of the map. Markers in bold were mapped in this study. Arrowheads with Ycen and Ytel show the directions towards the centromere and telomere, respectively.

The current BAC contig map of the horse MSY [37]–[38] comprises of 197 BAC clones which are tentatively arranged into 5 contigs (Fig. 2). In this study, 183 of these BACs were used as *selectors* (Table S2) in the cDNA selection procedure, to isolate expressed MSY sequences from testis. Primers for 29 of the MSY genes were amplified by PCR using DNA of each BAC clone as template, and the location of 26 genes in the 5 contigs was determined (Fig. 2). This confirmed our previous map data [37]–[38] and added 19 new genes to the map. Sequences of *ZNF33bY*, *ETY3* and *ETSTY6* were not present in the existing BACs, thus new clones were isolated from the BAC library. All new BACs mapped by FISH and STS content analysis (data not shown) to ECAY heterochromatin adjacent to contig I (Fig 2). Map locations of the 8 genes that were identified by STS content and BAC sequence analysis (see above) were known before. Therefore, the current gene map of horse MSY consists of 37 genes/transcripts that are assigned to 5 BAC contigs and the heterochromatic region (Fig. 2). The precise order of these markers, however, remains tentative, and will be ascertained once the contig map is complete.

### Gene copy numbers – cDNA FISH

The sensitivity of FISH is limited, therefore it is usually not possible to see signals produced by short (<1000 bp) cDNA sequences [44]–[45], unless these sequences are present in multiple copies. The average size of the cDNA sequences isolated in this study ranged between 300–850 bp. Most of the genes/transcripts that were mapped by STS content analysis to the multicopy region (Fig. 2) produced strong signals by cDNA FISH (Fig. 1c, d; Table 1), thus confirming the multicopy nature of these sequences. The only exception was *SRY* cDNA which was mapped in a BAC clone harboring multicopy genes *TSPY*, *RBMY* and *YIR2* but did not produce any signal by cDNA FISH. Therefore, we infer that *SRY* is a single copy gene in horse which is embedded within multicopy sequences. In contrast to the multicopy region, no FISH signals were observed with the cDNAs of genes located in the proximal part of contig I, and in contigs II–V (Fig. 2). Finally, the cDNA clones of *ETY3*, *ETSTY6* and *ZNF33bY* produced painting-like signals in ECAY and ECAXq21 heterochromatin (Fig. 1e, f) indicating that these sequences are highly amplified. Overall, we identified 15 ampliconic genes/transcripts – 12 in the multicopy region and 3 in ECAY heterochromatin (Fig. 2).

### Transcriptional activity and profiles of MSY genes in stallion testis

Direct selection yielded a range of cDNA sequences for different MSY genes indicating the relative transcriptional activity of these genes in stallion testis. For example, only one transcript was retrieved for *CUL4BY, ETY1-4, MT-ND1, NLGN4Y, RBMY, RFX5Y, RPS3AY, TMSB4Y,* and *YIR2*. In contrast, 54 and 43 cDNA sequences were found for *UBE1Y* and *SRY*, respectively (Table 1). We noticed that *SRY* was recovered in only those direct selection experiments where testis cDNA and Cot-1 ratio was low (1∶2). We re-analyzed the 1,420 bp sequence of the single coding exon [34] of *SRY* and discovered a 20 bp LTR repeat. This repeat probably anneals to Cot-1 DNA and eliminates *SRY* from selection experiments with high cDNA/Cot-1 ratios (1∶7.5). Further sequence analysis revealed that exonic LTR and/or simple repeats are present also in mouse, rat, rabbit, cat, dog and donkey *SRY* genes (Ensembl, http://www.ensembl.org/index.html; NCBI Entrez Nucleotide, http://www.ncbi.nlm.nih.gov/sites/entrez?db=nucleotide), and have not been reported before. The evolutionary as well as functional significance of these repeats in *SRY* coding sequence is yet to be explored.

Many transcripts were found also for *EIF3CY* (85) and *ZNF33bY* (64). However, these numbers might be inflated because the coding sequences of the autosomal and Y-derived homologs of the two genes were identical, and it was not possible to design RT-PCR primers to clearly distinguish between the autosomal and Y-derived cDNA amplicons.

**Figure 3:**
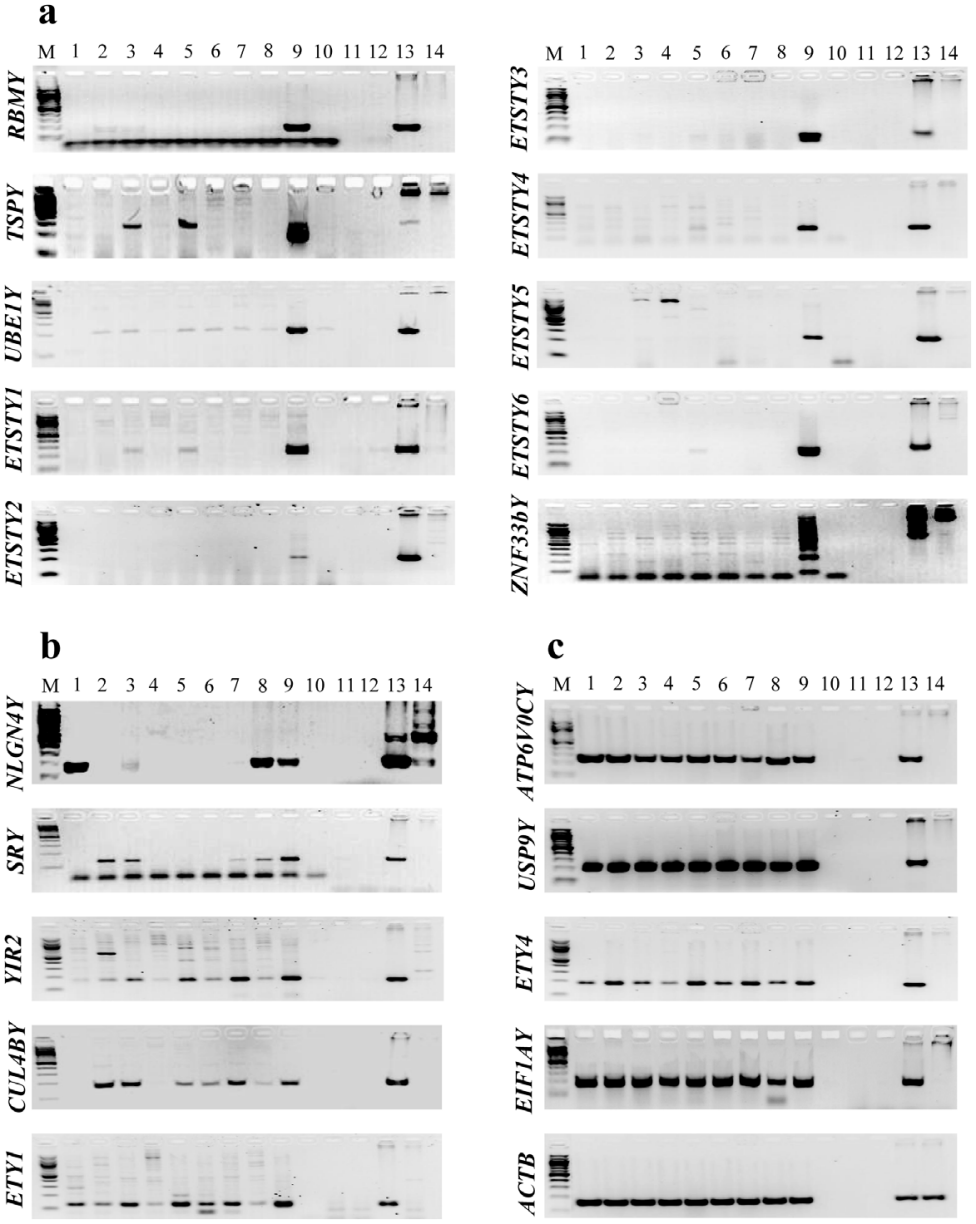
The results of RT-PCR (30 cycles) showing the expression of horse MSY genes in nine adult tissues. **a.** Testis-specific expression. **b.** Intermediate expression; **c.** Ubiquitous expression (*ACTB* was used as an internal control). Lanes: M - molecular markers (100 bp ladder, New England Biolabs); 1 - brain, 2 - kidney, 3 - heart, 4 – skeletal muscle, 5 - liver, 6 - lung, 7 - spleen, 8 - seminal vesicle, 9 - testis, 10 - no mRNA control, 11 - no RT control, 12 - no genomic DNA control, 13 - male genomic DNA control, 14 - female genomic DNA control.

Transcriptional profiles of the 37 MSY genes were studied on a panel of nine adult equine body tissues by reverse transcriptase (RT) PCR (Fig. 3). The primers for two MSY genes, *TBL1Y* and *STS-Y,* did not show male-specific amplification and hence were removed from analysis, leaving results for 35 genes. Transcription profiles of these 35 MSY genes/transcripts were classified in three categories (Table 1):

- Ten genes (*ETSTY1-6, RBMY, TSPY, UBE1Y, ZNF33bY*), all present in multiple copies (according to cDNA FISH), were expressed exclusively in testis (Fig. 3a);
- Six genes (*CUL4BY, ETY1, NLGN4Y, RFX5Y, SRY, YIR2*) showed intermediate or broader expression, and were transcribed in testis and in a few other tissues. Notably, *SRY* and *YIR2* expression was predominant in testis (Fig. 3b);
- Sixteen genes (*ATPV06CY*, *CYorf15, DDX3Y, EIF1AY, EIF2s3Y, EIF3CY, ETY2*, *ETY4, MT-ND1Y, MAP3K7IP3Y, RPS3AY, KDM5D, TMSB4Y, USP9Y, UTY, ZFY)* were expressed ubiquitously in all nine tissues studied (Fig. 3c).

The remaining three genes, *AMELY, KAL1Y* and *ETY3,* were not expressed in any of the 9 tissues. The results for *AMELY* were expected because the gene is known to be expressed exclusively in developing tooth buds [43], while *KAL1* might be a Y-linked pseudogene as it is in humans [1]. Transcriptional status of *ETY3*, however, remained tentative and will be ascertained in future studies using a more comprehensive panel of male equine adult and embryonic tissues. Until then, the marker is named as *E(T)Y3*.Testis-specific *ZNF33bY* and testis predominant *YIR2* produced several different RT-PCR amplicons (Fig. 3a, b) indicating that the multiple copies of the two genes give rise to many different transcripts in the horse tissues analyzed. The functional significance of multiple transcripts can be addressed in future studies.

Finally, it must be noted that since the tissue panel comprised of selected adult tissues, the specific expression profiles as determined in this study do not necessarily represent the functional profiles of the horse MSY genes throughout the development.

### Full-length cDNA sequences of multicopy, testis-specific MSY genes

Multicopy MSY genes with testis-specific expression are most frequently involved in male fertility related functions in human and mouse [1], [46]. Therefore, bearing in mind their possible role in stallion fertility, full length cDNA sequences were obtained for *TSPY* (1,037 bp), *ETSTY2* (2,323 bp) and *ETSTY5 (*1,635 bp). The length of horse *TSPY* cDNA is quite similar to human (1,160 bp; NM_003308) but half the size of the domestic cat (2,089 bp; clone a244, DQ329518) *TSPY*. Despite the size differences, the horse *TSPY* cDNA shares 78% sequence similarity with 522 nucleotides (nt) in human, and 75% similarity with 577 nt in cat *TSPY*. Due to the uncertainly of the RACE procedure, despite several attempts, only partially extended cDNA sequences were generated for *ETSTY1*, *3*, *4, 6*, and *RBMY*, while no additional sequence was obtained for *UBE1Y* and *ZNF33bY*. Novel transcripts *ETSTY1*, *3, 4* and *6* did not align with each other while using standard stringent assembly parameters in Sequencher program. When the sequence assembly parameters were relaxed, the three sequences did show some overlap. Thus, it is possible that they are members of a gene family which will be confirmed when the full sequence of the Y chromosome is available. The generation of full-length cDNA sequences was important to look for their protein coding ability and hence likely involvement in male fertility.

### Protein coding potential of MSY genes

To investigate how many MSY genes might have a protein coding potential, all cDNA sequences were analyzed for the presence of open reading frames (ORFs). We found ORFs in 18 horse MSY genes (Table 1). Among these, *TSPY* cDNA has eight ORFs of which the longest is 807 nucleotides. The remaining seven ORFs are present at different overlapping regions of the longest ORF and are 693 bp, 375 bp, 366 bp, 342 bp, 330 bp, 276 bp and 237 bp, respectively. All seven ORFs, except the shortest, encode *TSPY* protein of 269, 231, 125, 122, 114, 110 and 92 amino acids, respectively. The protein contains a NAP (nucleosome assembly protein) domain which is conserved across mammalian species, and is important for a diverse spectrum of cellular and molecular functions [47]. It is thus likely that TSPY protein can be important for male fertility related function in horses. Notably, ORFs were found in several novel, as yet equine-specific transcripts (Table 1) including the full length cDNA of *ETSTY2* and *ETSTY5*. The corresponding hypothetical proteins, however, showed no homology to known mammalian protein sequences. It is possible that novel equine transcripts encode novel proteins. Alternatively, these novel, as yet equine-specific amplified sequences, similarly to many ampliconic MSY sequences in human and chimpanzee [1], [4], might be non-coding transcription units.

### Comparative analysis of horse MSY genes in the donkey

There are striking differences in MSY gene content between some closely related species, such as human and chimpanzee [4] but not between others, like human and gorilla [24]. To investigate Y chromosome evolution in equids, we compared horse MSY gene content with that of the donkey (*Equus asinus*). Evolutionary distance between the two species is about 3 million years [48]–[49], thus half the time that separates human and chimpanzee [4].

We attempted PCR with the 37 horse MSY gene primers in donkey genomic DNA (Fig. S2), and showed that 29 genes, including 6 novel testis-specific transcripts (*ETSTY1-6*), are shared between the two equid Y chromosomes. Five genes (*DDX3Y, E(T)Y3, MAP3K7IP3Y, NLGN4Y, UBE1Y*) amplified in male and female donkeys, though in horses the same primers were male specific. Therefore, we were unable to confirm Y-specific nature of these sequences in donkeys. Similarly to the horse, it was not possible to distinguish by PCR between *STS-Y/X* and *TBL1Y/X* in donkeys. Remarkably, donkey amplicons of *E(T)Y3* and *ZNF33bY* were of different sizes compared to horse (Fig. S2), and *ETY2* and *EIFCY* (smaller male-specific sequence) were not found in the donkey.

**Figure 4:**
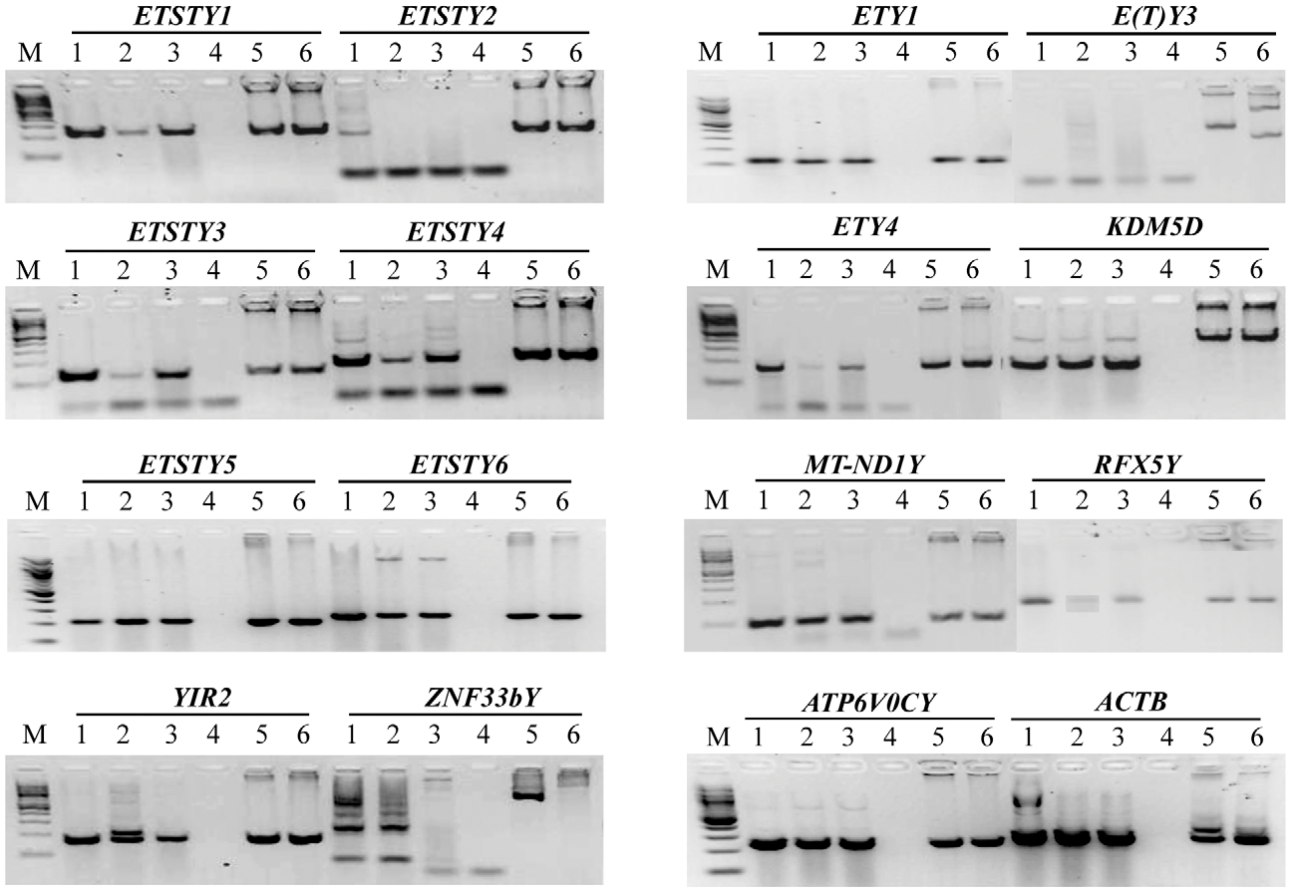
The results of RT-PCR (30 cycles) showing the expression of horse MSY genes in donkey testis. 1 - horse testis (positive control); 2–3 – testis of two different donkeys; 4 - no mRNA control; 5 - male horse genomic DNA control; 6 - male donkey genomic DNA control; M - molecular markers (100 bp ladder, New England Biolabs). *ACTB* was used as an internal control.

Next, we determined expression profiles of the 15 horse MSY genes, including all novel transcripts, five acquired sequences, and *KDM5D* as an X-degenerate control, in donkey testis (Fig. 4). All genes, except *E(T)Y3* and *ETSTY2*, were expressed in donkey and sequence identity between the homologous horse and donkey transcripts was 95-100% (data not shown). These data demonstrate that overall, the horse and donkey Y chromosomes are similar in DNA sequence and gene content, but show also species specific structural and transcriptional differences. More studies, including gene copy number analysis and detailed expression profiling of all asine MSY genes, will determine the homology between the two Y chromosomes in more detail.

### Comparison of horse MSY with other mammalian Y chromosomes

The two broad categories of mammalian MSY genes, X-degenerate and ampliconic are present in human [1], chimpanzee [50], gorilla [24], mouse [10], [51], cat [11]–[12], and cattle [13]. Additionally, Y chromosomes have frequently acquired sequences by transposition or retroposition from other parts of the genome [52]. In this study, we demonstrated that X-degenerate, ampliconic and acquired sequences are also present in the horse MSY (Table 2).

**Table 2:** Comparative status of horse MSY genes in other mammals.

Gene class	Gene symbol	Horse	Donkey	Human	Chimp	Gorilla	Cat	Mouse	Other mammals	References
X-degenerate	*AMELY*	SC	+	SC	SC	+	SC	-	SC- cattle, pig	[1], [4], [11], [13], [19], [24], [85]
	*CUL4BY*	MC	+	-	-	-	MC	-	MC-dog	[11]
	*CYorf15*	SC	+	SC	SC	+	MC	-	-	[1], [4], [11]–[12], [24], [85]
	*DDX3Y* *(DBY)*	SC	?	SC	SC	+	SC	SC	SC-cattle, pig, rat	[1], [4], [8], [11]–[12], [24], [85]
	*EIF1AY*	SC	+	SC	SC	+	SC	-	-	[1], [4], [11]–[12], [24], [85]
	*EIF2s3Y*	SC	+	-	-	-	SC	SC	SC-pig	[8], [11]–[12], [19]
	*KAL1Y*	SC	+	ps	-	-	-	-	PAR-cattle, pig, dog, sheep, goat	[1], [19], [58]
	*KDM5D*	SC	+	SC	SC	+	SC	SC	SC-dog, cattle, pig	[1], [4], [8], [11]–[12], [19], [24], [85]
	*MAP3K7IP3Y*	SC	?	-	-	-	-	-	-	this study
	*NLGN4Y*	SC	?	SC	SC	+	-	SC	PAR-cattle, dog, sheep, goat	[1], [4], [24], [58], [85]
	*RBMY*	MC	+	MC	MC	-	-	MC	-	[1], [4], [8]
	*SRY*	SC	+	SC	SC	+	MC	SC	SC-pig; MC-cattle?, rabbit, rat	[1], [4], [8], [11]–[14], [19], [24], [69], [85]
	*STS-Y*	SC	?	ps	ps	-	-	PAR	PAR-dog, cattle, pig	[1], [19], [86]–[87]
	*TBL1Y*	SC	?	SC	SC	+	-	-	PAR-cattle, dog, sheep, goat	[1], [4], [24], [58], [88]
	*TMSB4Y*	SC	+	SC	SC	+	-	-	-	[1], [4], [24], [85]
	*TSPY*	MC	+	MC	MC	-	MC	ps	MC-cattle, goat, sheep, pig, rat	[1], [4], [11], [13], [19], [56], [60]–[61], [89]
	*UBE1Y*	MC	?	-	-	-	MC	SC	rat, pig, SC	[8], [11]–[12], [19]
	*USP9Y*	SC	+	SC	SC	+	SC	SC	SC-pig, rat	[1], [4], [8], [11]–[12], [19], [24], [85]
	*UTY*	SC	+	SC	SC	SC	SC	SC	SC-pig	[1], [4], [8], [11]–[12], [19], [24], [85]
	*ZFY*	SC	+	SC	SC	+	SC	MC	SC-pig, rat	[1], [4], [8], [11]–[12], [24], [85]
Acquired	*ATP6V0CY*	SC	+	-	-	-	-	-	-	this study
	*EIF3CY*	SC	-	-	-	-	-	-	-	this study
	*MT-ND1Y*	SC	+	ps	-	-	-	-	-	this study
	*RFX5Y*	SC	+	-	-	-	-	-	-	this study
	*RPS3AY*	SC	+	-	-	-	-	-	-	this study
	*ZNF33bY*	HC	+	-	-	-	-	-	-	this study
Ampliconic	*YIR2*	SC	+	+	-	-	-	-	-	this study
	*ETSTY1*	MC	+	-	-	-	-	-	-	this study
	*ETSTY2*	MC	+	-	-	-	-	-	-	this study
	*ETSTY3*	MC	+	-	-	-	-	-	-	this study
	*ETSTY4*	MC	+	-	-	-	-	-	-	this study
	*ETSTY5*	MC	+	-	-	-	-	-	-	this study
	*ETSTY6*	HC	+	-	-	-	-	-	-	this study
	*ETY1*	MC	+	-	-	-	-	-	-	this study
	*ETY2*	SC	-	-	-	-	-	-	-	this study
	*E(T)Y3*	HC	+	-	-	-	-	-	-	this study
	*ETY4*	MC	+	-	-	-	-	-	-	this study

**SC** – single copy; **MC** – multicopy; **HC** – heterochromatic; **ps** – pseudogene; **+** - present; - not found; **PAR** –pseudoautosomal.

***The X-degenerate genes*** originate from mammalian proto-sex chromosomes and have been retained as gametologues on both the X and the Y chromosome [3], [12], [53]–[54]. They are typically orthologous between species, and are truly the only comparative loci across mammalian Y chromosomes. Indeed, almost two thirds of the 20 X-degenerate genes found in horse have a Y-linked homologue in primates [1], [4], [24], [55]–[56], carnivores [11]–[12], rodents [52], [57] or other mammalian species [13]–[14], [19], [58] (Table 2). Not surprisingly, all 20 genes are present in the donkey as well.

Like in other species [1], [11]–[12], the majority of horse X-degenerate genes are single copy sequences with broad or ubiquitous expression profiles (Table 1). A few X-degenerate genes, *viz.*, *CUL4BY, RBMY*, *TSPY* and *UBE1Y,* have been amplified and have acquired testis-specific or testis predominant expression (Table 1, Fig. 3a, b). Notably, *RBMY* is a multicopy testis-specific transcript in human, mouse and cat [1], [11], [59], and *TSPY* is a multicopy and testis-specific gene in most species studied so far [1], [11], [60]–[61]. The only known exception is mouse where *TSPY* has become a single copy pseudogene [62]. In comparison, more evolutionary changes have shaped *UBE1Y.* Horse is the only species where *UBE1Y* is both multicopy and with testis-specific expression (Table 1, Fig. 1a). Orthologs in other species (cat, pig, mouse) are single copy [11], [19], [63], and only murine *UBE1Y* is expressed exclusively in testis [64]. The gene has altogether been lost from human MSY [1], [65]. In mouse Y chromosome, *UBE1Y* is located in a region encoding the spermatogenesis factor, *Spy,* which is required for the normal proliferation of germ cells. Due to its testis-specific expression [64], the *UBE1Y* has been considered as a possible candidate gene regulating germ cell proliferation and, thus, male fertility. Given the high copy number and testis-specific transcription of *UBE1Y* in horses, we hypothesize that the gene has acquired functions that are specifically associated with ubiquitin activation and protein turnover [64] in equine germ cells. The high number of *UBE1Y* cDNAs recovered by direct selection (Table 1) also signifies the functional importance of these transcripts in horse testis.

An interesting finding was the presence of an equine Y-linked cullin 4B (*CUL4BY*) orthologue. This highly amplified and testis-specific transcript has as yet been identified in the cats and dogs [11]–[12]. The equine *CUL4BY* is also multicopy as in the two carnivores but has a broader expression profile being transcribed in testis, heart and kidney (Table 1). We speculate that the horse *CUL4BY* might be in a transitional stage towards restricting its E3 ubiquitin ligase functions [66] specifically to testis and male germ cell proliferation.

The most intriguing X-degenerate gene, however, is *SRY*. Considering the known function of *SRY* in sex determination at early stages of mammalian embryonic development [67], it comes as a surprise that the single copy *SRY* is transcribed at high levels in adult horse testis (Table 1). Functional importance of *SRY* in adult males is not yet known. However, the presence of multiple copies of *SRY* sequences in rabbit [68], rat [69] and cat [12] Y chromosomes further supports our assumption that *SRY* has more functions than sex determination, and the transcripts might be actively needed also in mature testis.

***Ampliconic sequences*** are defined as amplified or multiple segments of euchromatic sequences that exhibit as much as 99.9% identity over 10–100 kilobasepairs with other MSY sequences, and comprise multicopy gene families [1]. These regions have been sequenced only in human [1] and chimpanzee [4] though multicopy MSY gene families have been found also in other primates [54], mouse [7]–[8], carnivores [11], horse [37] and cattle [13]. The evolutionary origin of these sequences is diverse. Some genes, like *TSPY* or *RBMY*, originate from ancestral X-degenerate genes (discussed above). Others, like human *DAZ* and *CDY* [1], [70]–[71], mouse *RhoA* [72], cat *FLJ36031Y* [11], or horse *ZNF33bY* (this study) have been transposed and amplified from autosomes. Several ampliconic sequences, however, are Y-borne and show no sequence similarity between distantly related species [11]. Similarly, the horse MSY has acquired 10 novel amplified and expressed sequences that are partly shared with the donkey Y chromosome (in sequence homology and transcriptional status), but not with any other mammalian species studied so far (Table 2).

Unexpectedly, three horse cDNA sequences, *viz., E(T)Y3*, *ETSTY6* and *ZNF33bY* mapped to the ECAY heterochromatin. Two transcripts, *ETSTY6* and *ZNF33bY,* are expressed exclusively in testis (Table 1), and an ORF was found in the partially extended cDNA sequence of *ETSTY6*. To the best of our knowledge this is the second report that highly amplified transcripts with protein coding potential are found in mammalian Y chromosome heterochromatin. Previously, a 450 kb euchromatic island was identified in the pericentromeric heterochromation of the human Y chromosome [23]. Similarly to the horse, the region in humans is highly duplicated, and contains genes with ORFs including members of the homeobox gene family *DUX*. Most likely, these sequences in horse have been highly amplified to carry out important testicular function and are therefore expressed exclusively in testis.

Species-specific features were observed also in the distribution of ampliconic sequences in MSY. In horse, multicopy sequences are localized in relatively small regions in Contig I and the heterochromatin (Fig. 2) comprising two distinct blocks on ECAYq. Human amplicons are distributed between at least 5 distinct regions along HSAY [1], while in chimpanzee they are consolidated into two main blocks on Yp and proximal Yq [4]. In other species, such as mouse [9], cat [11] and pig (our unpublished data), tandemly repeated ampliconic arrays comprised of multicopy gene families are dispersed over the entire long arm of the Y chromosome. For example, some mouse testis-specific gene families (*Ssty, Asty*) each are present in as many as 65–100 copies [8], [11], [51] Thus, despite substantial differences, the architecture of horse MSY resembles more that of primates than other mammals studied so far.

Contrasting these structural differences in MSY multicopy gene families, functional features of these sequences tend to be more similar in different species. Like in human, mouse and cat [1], [4], [9], [11], most equine Y-borne amplified sequences are expressed exclusively or predominantly in testis (Table 1), and have presumably a role in testicular functions. Substantial evidences for this have been provided by human and mouse Y chromosome studies [7]–[8], [46], [51]. It has also been argued that gene amplification on sex chromosomes might be needed for rapid compensation for sex chromatin repression after male meiosis [26], [73]. Taken together, the multicopy portion of mammalian MSYs may share very little direct sequence homology between species, but is surprisingly consistent in function. Thus, the 10 multicopy and testis-specific genes identified in this study (Table 1) are the primary candidate genes for stallion fertility, and subject for future studies.

#### Acquired sequences

Acquisition of sequences from autosomes, the X-chromosome or mitochondrial genome is another characteristic feature of Y chromosome evolution [30], [54]. Such transposed or retrotransposed sequences can acquire testis-restricted functions, and are frequently amplified. The examples are human *DAZ* [1] or mouse *RhoA* [72] gene families. Other transposed sequences might lose their original functions and be retained as pseudogenes [1], [50]. Structural, functional, and evolutionary analyses of the three bovid Y specific gene families [15]–[16] also support the idea that the Y chromosome tends to acquire and amplify fertility related genes or even blocks of genes from other genomic regions [30]. Detailed molecular and functional analysis of bovine *DDX3Y* and it's X-linked and autosomal homologs show that the *bDDX3* gene family is expressed predominantly in testis and brain, thus being a good candidate to be involved in spermatogenesis [74]. Such gene traffic, however, is species or group specific because in different species different autosomal genes have acquired Y-linked counterparts. This is consistent with the results of the present study showing that none of the autosome derived genes on horse MSY have, as yet, been found Y-linked in other mammals (Table 2). Among the six acquired genes on the horse MSY, only *ZNF33bY,* a member of zinc finger protein families, has been amplified and become testis-specific. Likewise, two zinc finger genes, *ZNF280BY* and *ZNF280AY,* have been acquired by the cattle Y chromosome [16]. Similarly to the equine *ZNF33bY*, the cattle *ZNF280BY* is predominantly expressed in testis and possible involvement of this gene in spermatogenesis has been suggested [16]. The remaining five acquired equine MSY genes were single copy genes with intermediate or ubiquitous expression, and their functional importance has yet to be determined. We hypothesize that the intermediately expressed *RFX5Y* might be associated with minor histocompatibility complex and H–Y antigens [75] because the autosomal RFX5 protein is a part of conserved transcriptional coactivator complex binding to the MHC-II promoters [76]. The ubiquitously expressed male specific homolog of mitochondrial *MT-ND1* is probably the first NUMT (nuclear sequences of mitochondrial origin) found in the horse MSY. Though limited data are available for NUMTs in the horse genome [77], human studies show that the Y chromosome is more susceptible for mtDNA insertions than the rest of the genome [78]. Next, given the known role of the autosomal *ATP6V0C*, a component of a multi-subunit membrane transporter, in regulating sperm motility and maturation in humans [79], it is possible that the equine Y-linked *ATP6V0CY* has acquired a similar function. Finally, we could not clearly determine the transcriptional status of *EIF3CY* and *RPS3AY* because the RT-PCR amplicons of Y-linked and autosomal homologs were the same size. Therefore it is likely that *EIF3CY* and *RPS3AY* are pseudogenes and the recovered transcripts originate from their autosomal counterparts. Overall, we conclude that gene acquisition on the Y chromosome is a species or lineage specific event, and the presence of equine acquired Y-linked sequences was observed only in the donkey but not in other mammals studied to date (Table 2).

### Concluding remarks

We have demonstrated that ECAY, like the Y chromosomes in primates, mouse, cattle and cat, is comprised of functional genes and expressed sequences. About half of horse MSY genes are shared with other mammals and are of ancestral origin. The remaining genes on the MSY comprise novel Y-borne or transposed genes which, according to the current comparative information, are horse and/or donkey specific. All Y-borne novel sequences and *ZNF33bY* have been amplified and became multicopy on ECAY. The horse MSY gene catalogue is the first detailed information of a Y chromosome in a perissodactyl species, thus improving our knowledge about the evolutionary processes that have shaped Y chromosomes in different eutherian lineages. We infer that despite substantial differences in the organization of mammalian Y chromosomes, the likely functions of several MSY genes and transcripts might be conserved and are related predominantly to testis and possibly to male fertility. These findings establish an important foundation for the study of Y-linked genetic factors governing fertility in stallions. Finally, since the genome sequencing project of the domestic horse used DNA from a female animal, the MSY gene catalogue is the first extensive collection of male specific genes and sequences in horses.

## Materials and Methods

### Ethics Statement

Procurement of equine and asine tissues was performed according to the *United States Government Principles for the Utilization and Care of Vertebrate Animals Used in Testing, Research and Training* and were approved by the Clinical Research Review Committee (CRRC #08-33) at Texas A&M University.

### DNA samples and chromosome preparations

Genomic DNA of five normal male and female horses, and one female and two male donkeys was isolated from peripheral blood using standard protocols [80]. Equine chromosome preparations were obtained from blood lymphocytes of a male horse following our protocol [81].

### Selection of Y-specific cDNA sequences

Two rounds of direct cDNA selection were carried out as described earlier [82]. We used normal adult horse testis cDNA as the *driver*, and horse Y chromosome sequences as *selectors*. Testis mRNA was isolated using Fast Track 2.0 mRNA isolation kit (Invitrogen), and cDNA was synthesized using random primers and Superscript II reverse transcriptase (Invitrogen). The cDNA was adapter-ligated and amplified by PCR. Horse Y chromosome specific *selectors* were generated from: i) 7000 copies of flow sorted and GenomiPhi (Amersham Biosciences) amplified horse Y chromosome (provided by Cambridge Resource Centre for Comparative Genomics), and ii) 183 BAC clones from the horse MSY contig map [37] (Fig. 2, Table S2). The BAC clones were divided into 6 pools with ∼30 clones in each. Amplified testis cDNA was annealed with horse Cot-1 DNA (cDNA/Cot-1 ratio ranged from 1∶2 to 1∶7.5) for 4 h to block repetitive sequences. In some experiments, we added *UBE1Y* cDNA to Cot-1 DNA to conceal this most abundant Y-specific transcript, and facilitate the discovery of unique expressed sequences. Flow sorted ECAY and the 6 MSY BAC pools were labeled with biotin according to our protocol [81], and hybridized individually with pre-annealed testis cDNA for 50 h. Testis cDNA and biotinylated Y chromosome hybrids were selected with streptavidin coated paramagnetic beads (Dynabeads® M-280 Streptavidin, Invitrogen) and Y-specific testis cDNA was eluted. This primary selected cDNA was amplified by PCR and used for a second round of hybridization as described above.

### Cloning, sequencing and analysis of cDNA sequences

Selected cDNA from the second round of hybridization was amplified by PCR and cloned *en masse* into TOPO-TA cloning vector (Invitrogen). Plasmid clones were picked and grown overnight at 37°C in 96-well culture plates containing LB media and ampicillin (50 µg/ml). Plasmid DNA was isolated by alkaline lysis using REAL-prep96 kit (Qiagen). Randomly selected 152 plasmid clones were digested with *EcoRI* (Invitrogen), and analyzed on 2% agarose gels for the presence of inserts. The cDNA clones were sequenced using BigDye (Applied Biosystems) terminator chemistry, universal primers and ∼300-500 ng of plasmid DNA as a template. The sequencing reactions were resolved on an ABI-3730 capillary sequencer (Applied Biosystems). Sequences were quality trimmed and assembled into contigs using Sequencher V 4.7 software (GeneCodes Co). The contigs were checked for repetitive elements with RepeatMasker (http://www.repeatmasker.org) and analyzed using Discontiguous MegaBLAST (http://www.ncbi.nlm.nih.gov/BLAST/Blast.cgi; cut-off threshold 1e-8) to identify putative orthologs in human, mouse and other mammalian genomes. The sequences were aligned with WG sequence assembly *EquCab2* of a female horse (http://www.ensembl.org/Equus_caballus/index.html), and likely male-specific cDNAs were subtracted. Male specificity of the cDNA sequences was further validated by PCR by using cDNA-specific primers (see below) on male and female genomic DNAs. Exon-intron boundaries of the partial cDNA sequences were tentatively determined using orthologous sequences of mammalian Y-linked genes (http://www.ensembl.org/index.html), and exonic primers for PCR (Table S1) were designed using Primer 3 software (http://frodo.wi.mit.edu/primer3/input.htm). Where possible, the primers were designed in neighboring exons to span an intron. In most cases, however, intron spanning primers could not be designed because of limited sequence information and knowledge about the gene structure. All PCR reactions were carried out in 10 µl volume containing 1X PCR buffer (Sigma Aldrich), 0.3 pmol of each primer, 0.2 mM dNTPs, 1.5 mM MgCl_2_, 0.25 units JumpStart REDTaq DNA polymerase (Sigma Aldrich), and 50 ng of genomic DNA. Each primer pair was amplified from the genomic DNA of 5 normal male and 5 normal female horses, and from one female and two male donkeys. The PCR products were stained with ethidium bromide and resolved on 2.0 % agarose gels.

### Sequence tagged site (STS) content and BAC end sequence analysis

Male specific cDNA sequences were assigned to MSY contig map by STS content analysis. The presence or absence of cDNA sequences in MSY BAC clones was determined by PCR using exonic cDNA primers and DNA from each of the 183 MSY BAC clones. If a male specific cDNA sequence was not found in the MSY contig map, the cDNA primers were used to screen the CHORI-241 library by PCR. New BAC clones were isolated as described by us earlier [38]. BAC end sequences were retrieved from NCBI (http://www.ncbi.nlm.nih.gov/) Entrez Nucleotide and analyzed by NCBI BLAST. Additionally, 44 anonymous male-female specific cDNA sequences were PCR amplified on the 12 BAC clones that form the minimum tiling path of the horse PAR [38]. One gene, *ATP6V0CY*, was discovered by analyzing the whole sequence of the BAC 107.3H9 [42].

### Fluorescence *in situ* hybridization (FISH)

We used FISH to check the Y-specificity of flow sorted ECAY, the 6 MSY BAC pools, all newly isolated BAC clones, and to evaluate copy numbers of male specific cDNA sequences. Probe labeling, *in situ* hybridization, signal detection and image analysis were carried out according to our detailed protocol [81].

### Tissue collection, RNA isolation and reverse transcriptase (RT)-PCR

Fresh necropsy samples of normal adult male horse tissues, *viz*., brain, kidney, heart, skeletal muscle, liver, lungs, spleen, seminal vesicle, and testes, were collected in RNA-later (Ambion). Likewise, testis tissue was obtained by castration from two male donkeys. Tissue specific mRNA was extracted with FastTrack 2.0 Kit (Invitrogen). If it was possible to predict the gene structure and exon-intron boundaries, intron-spanning primers were designed from neighboring exons for RT-PCR. Otherwise, and in cases where introns were very large, exonic primers (see above) were used. Primers and PCR conditions are listed in Table S1. RT-PCR reactions were carried out in 15 µl volume using Superscript III One-Step RT-PCR System and Platinum Taq DNA polymerase (Invitrogen), 40 pmol of each primer and 10 ng of mRNA. The cycling conditions were as follows: 30 min at 50°C, 2 min at 94°C, 30 cycles of 15 s at 94°C, 30 s at 58°C, 1 min at 68°C, and final extension at 68°C for 5 min. Genomic controls were run simultaneously with the mRNA samples and RT-PCR products were visualized on 2.0% agarose gels. In order to compare the gene expression among the selected tissues, a housekeeping gene *ACTB* was used as an internal control.

### Generation of full-length cDNA using 3′ and 5′ RACE

Rapid Amplification of cDNA Ends (RACE) was performed separately for 3′ and 5′ ends using GeneRacer^TA^ kit (Invitrogen) and 1 µg of testis total RNA. For the 5′ end of each partial cDNA sequence, one 5′ reverse primer and one 5′ nested reverse primer were designed using Primer 3 software (http://frodo.wi.mit.edu/primer3/input.htm) in conjunction with GeneRacer^TA^ 5′ forward and 5′ nested forward primers, respectively (Table S3). Similarly, 3′ forward primer and one 3′ nested forward primer were designed in conjunction with GeneRacer^TA^ 3′ reverse and 3′ nested reverse primers, respectively (Table S3). In both cases, manufacturer's instructions were followed. The first round PCR cycling conditions for RACE were: hot-start at 94°C for 2 min; 5 cycles of 94°C for 30 s, 72°C for 1 min; 5 cycles of 94°C for 30 s, 70°C for 1 min; 20 cycles of 94°C for 30 s, 65°C for 30 s, and 68°C for 1 min. A final extension of 10 min at 68°C completed the reaction. The PCR product obtained from the first round of amplification was diluted 10 times and 1 µl of the dilution was used as a template for nested RACE PCR with nested primers. Nested RACE PCR cycling conditions were as follows: hot-start at 94°C for 2 min; 20 cycles of 94°C for 30 s, 65°C for 30 s, and 68°C for 2 min, followed by final extension of 10 min at 68°C. Products of nested RACE PCR were resolved on a 2% agarose gel, the bands were cut, and PCR products were eluted using S.N.A.P. columns provided with the kit. PCR products were cloned using TOPO TA Cloning Kit for Sequencing (Invitrogen). Transformed cells were plated on LB agar containing 50 µg/ml of ampicillin. Colonies were picked after an overnight incubation at 37°C and cultured overnight at 37°C in LB medium with 50 µg/ml ampicillin. Plasmid DNA was extracted using REAL Prep 96-well Kit (Qiagen), and sequenced with universal primers as described above.

### Open Reading frames and potential proteins

All cDNA sequences, full-length and partial, were analyzed for the presence of open reading frames (ORFs) using Sequencher V 4.7 (GeneCodes Co) and NCBI ORF finder (http://www.ncbi.nlm.nih.gov/projects/gorf/) softwares packages. The potential protein sequences were compared with the available protein database using NCBI protein BLAST algorithm BLASTP (http://blast.ncbi.nlm.nih.gov/Blast.cgi). Additionally, NCBI conserved domain database search (http://www.ncbi.nlm.nih.gov/Structure/cdd/wrpsb.cgi) was used to identify the presence of any conserved domain in the putative protein sequences.

## Supporting Information

Figure S1 **Amplification by PCR (30 cycles) of** ***EIF3CY*** **(lanes 1–2),** ***RPS3AY*** **(lanes 3–4) and** ***ZNF33bY*** **(lanes 5–6) from male (odd numbered lanes) and female (even numbered lanes) horse genomic DNA.** Note that the lower bands are present only in males. M1 - molecular markers (100 bp ladder, New England Biolabs), M2 (1kb ladder, New England Biolabs).
(TIF)

Figure S2 **Comparative amplification by PCR (30 cycles) of horse MSY genes from horse and donkey genomic DNA.** 1 – male horse; 2–3 – two male donkeys; 4- female donkey; M - molecular markers (100 bp ladder, New England Biolabs).
(TIF)

Table S1 **In formation about horse MSY genes, PCR primers, and sequences**
(DOC)

Table S2 **List of BAC clones used for cDNA selection**
(DOC)

Table S3 **Primer for 3′ and 5′ RACE PCR**
(DOC)
