# Supplementary figures and images for: A Gene Catalogue of the Euchromatic Male-Specific Region of the Horse Y Chromosome: Comparison with Human and Other Mammals
Source: PLoS One. 2011 Jul 25;6(7):e21374. doi: 10.1371/journal.pone.0021374 (PMC3143126; doi:10.1371/journal.pone.0021374)

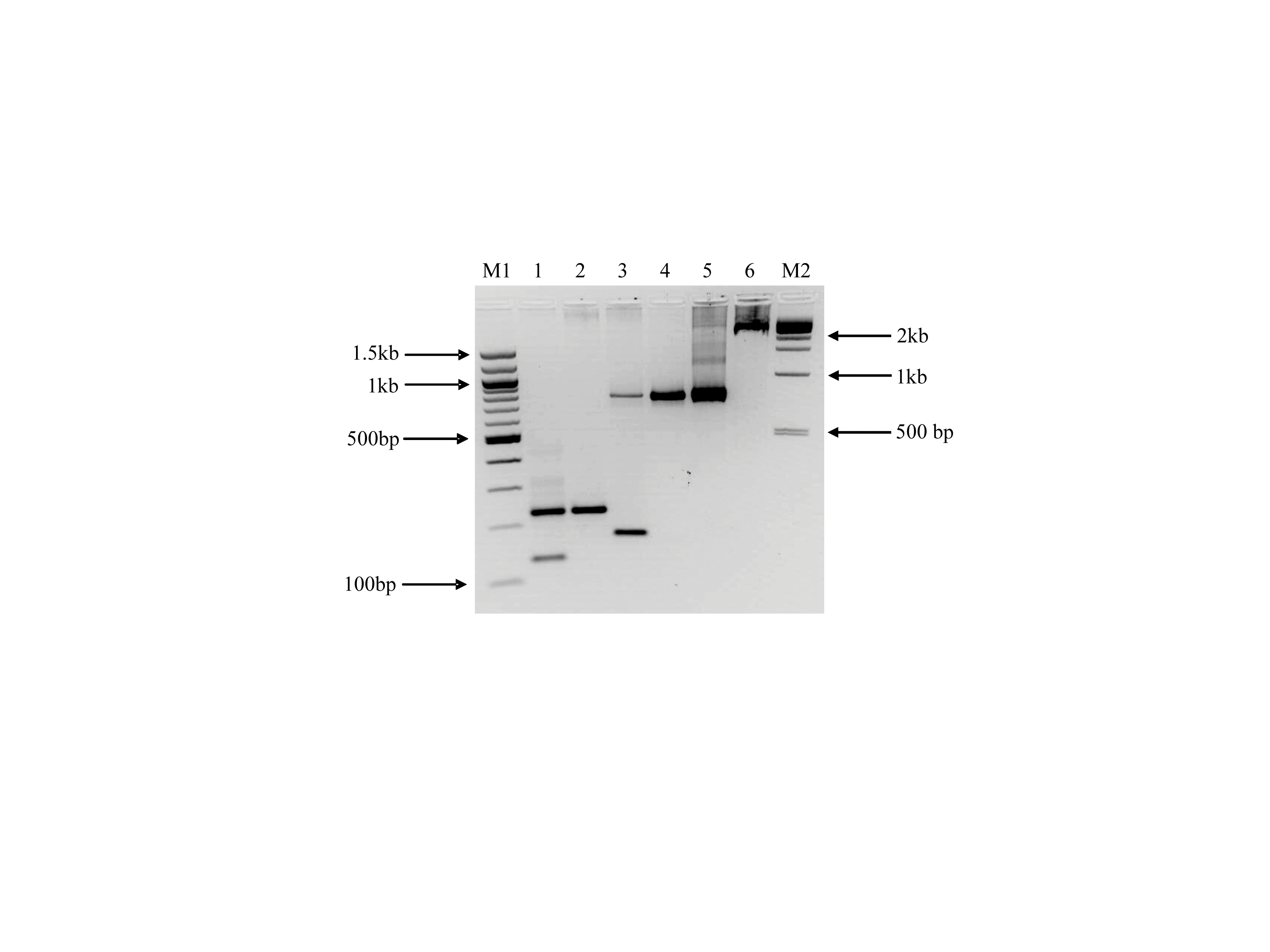

Supplement: Figure S1 — Amplification by PCR (30 cycles) of EIF3CY (lanes 1–2), RPS3AY (lanes 3–4) and ZNF33bY (lanes 5–6) from male (odd numbered lanes) and female (even numbered lanes) horse genomic DNA. Note that the lower bands are present only in males. M1 - molecular markers (100 bp ladder, New England Biolabs), M2 (1kb ladder, New England Biolabs). (TIF) [file pone.0021374.s001.tif]

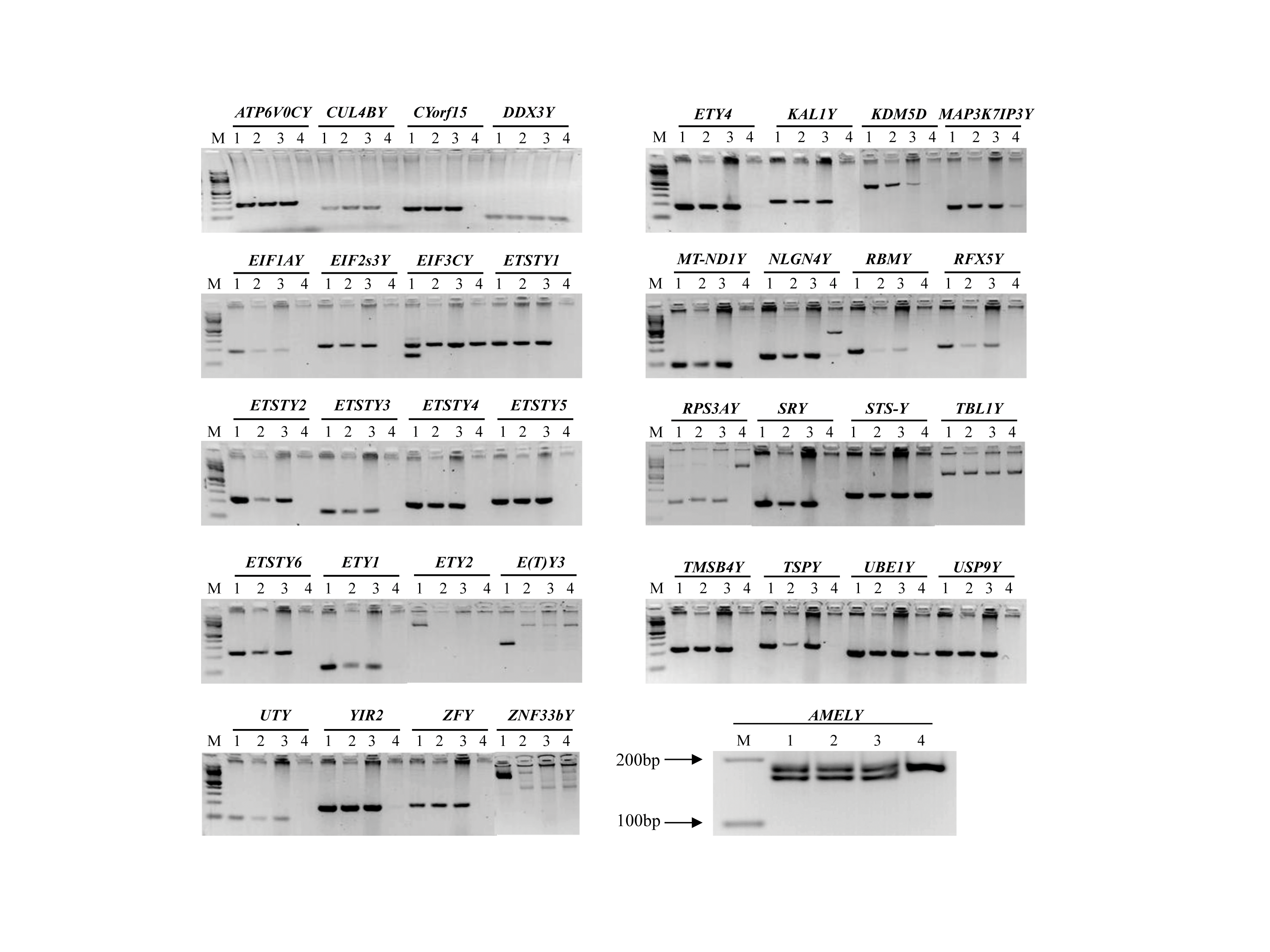

Supplement: Figure S2 — Comparative amplification by PCR (30 cycles) of horse MSY genes from horse and donkey genomic DNA. 1 – male horse; 2–3 – two male donkeys; 4- female donkey; M - molecular markers (100 bp ladder, New England Biolabs). (TIF) [file pone.0021374.s002.tif]
