# Supplementary material for: A Gene Catalogue of the Euchromatic Male-Specific Region of the Horse Y Chromosome: Comparison with Human and Other Mammals
Source: PLoS One. 2011 Jul 25;6(7):e21374. doi: 10.1371/journal.pone.0021374 (PMC3143126; doi:10.1371/journal.pone.0021374)
Supplement: Table S1 — In formation about horse MSY genes, PCR primers, and sequences (DOC) [file pone.0021374.s003.doc]

**Table S1. Information about horse MSY genes, PCR primers, and sequences**

| **Gene symbol** | **Gene name** | **Primers 5′ to 3′** | **Ta, °C** | **Genomicsproduct size, bp** | **cDNA**  **product size, bp** | **GenBank accession**  **No.** |
| --- | --- | --- | --- | --- | --- | --- |
| **AMELY* | Amelogenin, Y-linked | F:CCAACCCAACACCACCAGCCAA  ACCTCCCT  R:AGCATAGGGGGCAAGGGCTGCA AGGGGAAT | 65 | 160 M,  184 M/F | same | AB032194 |
| **ATP6V0CY* | ATPase, H+ transporting, lysosomal 16kDa, V0 subunit c | F:TAGAGGCCAAGGACCTCAGA  R:TGGCATCATCGCTATCTACG | 58 | 246 | same | n/a |
| *CUL4BY* | Cullin 4 B Y | F: TGTGGGGTTCGTGTGAAATA  R: CAAGGATCGCTGGGTCTTAC | 58 | 172 | same | EU687546 |
| *CYorf15* | Chromosome Y open reading frame 15 | gDNA primers F:CAACCATGCATTGAAAGAGG  R:TGCACTCCGATTCTTGTTGA  Intron-spanning primers F:CTAGGTGGCGACGCAAGTGA  R:TGCACTCCGATTCTTGTTGA | 58 | 152 | 358 | EU687545 |
| *DDX3Y* | DEAD (Asp-Glu-Ala-Asp) box polypeptide 3, Y-linked | gDNA primers  F:CTCGAGATCCAAAACTGCTG  R:TGATAAAAACAGTTCAGGGTGGA  Intron spanning primers F:CTCGAGATCCAAAACTGCTG R:GCTGGTCTGGACCTGAACTC | 58 | 68 | 181 | EU687547 |
| *EIF1AY* | Translation initiation factor 1A Y | F:GATCGTGGCCTTCTGACATT  R:TTATTTTTGGGCATGGTGGT | 58 | 187 | same | ET052957 |
| **EIF2s3Y* | Eukaryotic translation initiation factor 2, subunit 3 gamma, Y- linked | F:GAGCCATCTGTGTGATCGTC  R:TATTCCTGGCCCTAAGCACA | 58 | 223 | same | BV140834 |
| *EIF3CY* | Eukaryotic translation initiation factor 3, subunit C on Y | F:CCCAAGCAGGGTACCTATGG  R:GGACAGAAGTGACGCAATCA | 58 | 134 M, 230 M/F | same | EU687548 |
| *ETSTY1* | Equus Testis-specific transcript Y1 | F:GACGGACGACCTTGTGTTTT  R:CTAGTGGCGAGTCCTTTTGG | 58 | 234 | same | EU687549 |
| *ETSTY2* | Equus Testis-specific transcript Y2 | F:ATCATCGTGGAAAGCCTCAC  R:AGTGCTGAAGAGGCTGTGGT | 58 | 223 | same | EU687550 |
| *ETSTY3* | Equus Testis-specific transcript Y3 | F:TTACATTTGTTGCGCCATGT  R:GCCCAAAGAAGTAACCGACA | 58 | 134 | same | EU687551 |
| *ETSTY4* | Equus Testis-specific transcript Y4 | F:GCGTCTGTGCAGATGTGTCT  R:GCTCATGCAGTCAAACAGGA | 58 | 175 | same | EU687552 |
| *ETSTY5* | Equus Testis-specific transcript Y5 | F:CAAAACCAAGAGGAGGACCA  R:CTCCAGAGGCAGGTACTTCG | 58 | 210 | same | EU687553 |
| *ETSTY6* | Equus Testis-specific transcript Y6 | F:ACATGGCGCAACAAATGTAA  R:TAGCTGTTTGCTGCAGTGCT | 58 | 245 | same | EU687554 |
| *ETY1* | Equus transcript Y1 | F:TCCAGAGCAACAACAGCAAC  R:CATCAGTCTGCCCAAACCTT | 58 | 127 | same | EU687555 |
| *ETY2* | Equus transcript Y2 | F:TAAGGCTTCCCTCCTCCAAT  R:CCAGTGACCCGACATACTGA | 58 | 850 | same | EU687556 |
| *E(T)Y3* | Equus (transcript) Y3 | F:TTTTGGCTTGTGTCTTTCTCTG  R:ATAGGGCCAGACTTTCACAGC | 58 | 150 | same | EU687557 |
| *ETY4* | Equus transcript Y4 | F:TGGGGATATTGGCTTAGCTG  R:CTGGGAGCACGTCTGTATCA | 58 | 180 | same | EU687558 |
| **KAL1Y* | Kallmann Syndrome 1 on Y | F:AGGCACAGTCTTAGGGCAAA  R: TTTTGGCATTCCCTTCTCTG | 58 | 231 | same | CU091759 |
| *KDM5D (SMCY)* | lysine (K)-specific demethylase 5D | F: AACAGCGAGCCAATGTTTTT  R: GCAAAATTCTGGGAAATCCA | 58 | 400 | same | EU687564 |
| **MAP3K7-IP3Y* | Mitogen-activated protein kinase kinase kinase 7 interacting protein 3 on Y | F: GTGGAATCCCTATTGCTAAAGTT  AC  R: CCAGAGAGCTGTGACCAAG | 58 | 138 | same | CU028713 |
| *MT-ND1Y* | Mitochondrially encoded NADH dehydrogenase 1 on Y | F:CCCTCCGCTTTCCTAGACC  R:CAACGATGGCTTGAAAGGAT | 58 | 100 | same | EU687559 |
| *NLGN4Y* | Neuroligin 4 isoform Y | F:GGGGATCCATCTTTGTGTTG  R:GTCACACAGCAGGCTCTGAC | 58 | 156 | same | EU687560 |
| *RBMY* | RNA-binding motif Y | F:TTCGGCCTTCTCTTTCACAT  R:ACTCAAGCAGCCGAAATGAT | 58 | 180 | same | EU687561 |
| *RFX5Y* | Regulatory factor X 5 on Y | F:ACCCTTAGGGGGAAAAATCC  R:TTTCGTCCCTCAAGTTCCTG | 58 | 201 | same | EU687562 |
| *RPS3AY* | Ribosomal Protein S3A | F:CCGGAAGAAGATGATGGAAA  R:CAAACTTGGGCTTCTTCAGC | 58 | 179 M, 790 M/F | same | EU687563 |
| *SRY* | Sex determining region Y | F:TGCATTCATGGTGTGGTCTC  R:ATGGCAATTTTTCGGCTTC | 58 | 200 | same | EU687565 |
| **STS-Y* | Steroid sulfatase (microsomal), isozyme S on Y | F:TGTGTGTTTCTGTCATGGGGATTACATC  R:CAGACAATGTTTCCCAGTGACAATTGATTA | 58 | 210 M/F | n/a | AF133205 |
| **TBL1Y* | transducin (beta)-like 1, Y-linked | F: CACTCGAAACCAATGGAA  R: TTCCATATCCTGGCAGTCGA | 50 | 500 M/F | n/a | CT967953, CT967954 |
| *TMSB4Y* | Thymosin (beta) 4 Y | gDNA primers  F:ACCCACCCAGCCTCTTACTT  R:GCCTAAGCTGCCAATATCCA  Intron spanning primers F:ACCCACCCAGCCTCTTACTT  R:TTGAAGAAGACGGAAACGC | 58 | 246 | 334 | EU687566 |
| *TSPY* | Testis-specific Protein Y | F: GAAGTCAGGCACACCAGTGA  R: TAAGGCTGCAGTTGTCATGC | 58 | 280 | 189 | EU687567 |
| *UBE1Y* | Ubiquitin activating enzyme Y | F:TGGCCAACTCACGGCTGATCCAA  R:CTTCTCCACTCACCCTACTTGGG | 58 | 210 | same | EU687568 |
| *USP9Y* | Ubiquitin-specific protease 9 Y | F:GGTTATGAAATGGTCTCTGC  R:CGAGTCTGTCCATCAGGAGTC | 58 | 228 | same | EU687569 |
| **UTY* | Ubiquitously transcribed tetratricopeptide repeat gene, Y-linked | F: CAGCTGTTTTCGGTGATGAG  R: GCCTCCTTCTCTTCGGTTG | 54 | 110 | same | CU148483 |
| *YIR2* | Inverted repeat 2 Y | F:AGGGTTGGGCTAAGTCACCT  R:ACCTTGGATCCAGACTCACG | 58 | 170 | same | EU687570 |
| *ZFY* | Zinc finger Y | F:TGAGCTATGCTGACAAAAGGTG  R:TCTTTCCCTTGTCTTGCTTGA | 58 | 186 | same | EU687571 |
| *ZNF33bY* | Zinc Finger protein 33b on Y | F:CCACAGCAAATACAGGAGCA  R:GTCTGACTCCTCCCCCTTTC | 58 | 800 M,  3000 M/F | 242 | EU687572 |

* - genes identified only from MSY BAC clones; M – male; M/F – male and female, Ta – annealing temperature.
