# Supplementary material for: A Gene Catalogue of the Euchromatic Male-Specific Region of the Horse Y Chromosome: Comparison with Human and Other Mammals
Source: PLoS One. 2011 Jul 25;6(7):e21374. doi: 10.1371/journal.pone.0021374 (PMC3143126; doi:10.1371/journal.pone.0021374)
Supplement: Table S2 — List of BAC clones used for cDNA selection (DOC) [file pone.0021374.s004.doc]

**Table S2. List of BAC clones used for cDNA selection**

| **BAC** | **Contig** | **Library** |
| --- | --- | --- |
| 002 E4 | IV | CHORI241 |
| 003A5 | III | CHORI241 |
| 005.2A8 | IV | TAMU |
| 006B10 | III | CHORI241 |
| 008.3G9 | III | TAMU |
| 011B8 | III | CHORI241 |
| 012.2 E5 | II | TAMU |
| 012G3 | III | CHORI241 |
| 013 E2 | II | CHORI241 |
| 015.2 E9 | II | TAMU |
| 015.2A9 | II | TAMU |
| 016.4C5 | II | TAMU |
| 017.2C11 | III | TAMU |
| 017D15 | I multicopy | CHORI241 |
| 018K2 | III | CHORI241 |
| 019D21 | II | CHORI241 |
| 020.1G12 | I multicopy | TAMU |
| 020B4 | II | CHORI241 |
| 020L18 | IV | CHORI241 |
| 022.4 E3 | I multicopy | TAMU |
| 022G3 | V+PAR | CHORI241 |
| 022P7 | I | CHORI241 |
| 024.4G8 | I multicopy | TAMU |
| 024I23 | I multicopy | CHORI241 |
| 026.4A6 | II | TAMU |
| 026B21 | I multicopy | CHORI241 |
| 027.1A2 | I multicopy | TAMU |
| 027A12 | III | CHORI241 |
| 027B13 | III | CHORI241 |
| 03.4H8 | I + PAR | TAMU |
| 032H24 | I + PAR | CHORI241 |
| 032K15 | I + PAR | CHORI241 |
| 034 E15 | I + PAR | CHORI241 |
| 037.4D11 | I multicopy | TAMU |
| 037D2 | II | CHORI241 |
| 039P6 | III | CHORI241 |
| 042.4B5 | I | TAMU |
| 047.1H3 | IV | TAMU |
| 047.2A3 | IV | CHORI241 |
| 047B7 | II | CHORI241 |
| 049.2F10 | I | TAMU |
| 049.3F11 | I | TAMU |
| 049J16 | I | CHORI241 |
| 052H5 | I multicopy | CHORI241 |
| 054F13 | I | CHORI241 |
| 054J7 | I multicopy | CHORI241 |
| 055N19 | V | CHORI241 |
| 060D8 | I | CHORI241 |
| 061.4H8 | I + PAR | TAMU |
| 061G21 | II | CHORI241 |
| 061J18 | II | CHORI241 |
| 063H12 | V+PAR | CHORI241 |
| 063I4 | II | CHORI241 |
| 064P16 | I multicopy | CHORI241 |
| 066M24 | III | CHORI241 |
| 067.1G8 | III | TAMU |
| 067.4G1 | I multicopy | TAMU |
| 070F17 | II | CHORI241 |
| 072G23 | III | CHORI241 |
| 072G7 | I multicopy | CHORI241 |
| 074P12 | V+PAR | CHORI241 |
| 077M19 | I multicopy | CHORI241 |
| 079.4H1 | I | TAMU |
| 080.4F7 | I + PAR | TAMU |
| 081F24 | I multicopy | CHORI241 |
| 081F8 | III | CHORI241 |
| 083H5 | IV | CHORI241 |
| 086.2F8 | II | TAMU |
| 086J1 | I multicopy | CHORI241 |
| 087.3A5 | II | TAMU |
| 089.3B11 | IV | TAMU |
| 090B11 | IV | CHORI241 |
| 090G18 | II | CHORI241 |
| 090P8 | I multicopy | CHORI241 |
| 091.4G10 | III | TAMU |
| 095.4B8 | II | TAMU |
| 095.4F10 | I + PAR | TAMU |
| 097D2 | II | CHORI241 |
| 100.3A11 | IV | TAMU |
| 100.4F5 | IV | TAMU |
| 100H13 | I + PAR | CHORI241 |
| 101H8 | II | CHORI241 |
| 102J15 | IV | CHORI241 |
| 103.3A6 | I multicopy | TAMU |
| 106F1 | V | CHORI241 |
| 106J17 | III | CHORI241 |
| 107.3H9 | I | TAMU |
| 108.4C7 | II | TAMU |
| 110.3H12 | V | TAMU |
| 111.2F5 | I multicopy | TAMU |
| 112 E12 | III | CHORI241 |
| 112.1A9 | V | TAMU |
| 112C10 | I multicopy | CHORI241 |
| 114 E24 | III | CHORI241 |
| 114I17 | II | CHORI241 |
| 117.4F7 | II | TAMU |
| 118.1A9 | III | TAMU |
| 118L7 | II | CHORI241 |
| 118N21 | III | CHORI241 |
| 119K22 | II | CHORI241 |
| 120.1A5 | IV | TAMU |
| 120A19 | II | CHORI241 |
| 121G24 | IV | CHORI241 |
| 121H9 | II | CHORI241 |
| 124.3G9 | V | TAMU |
| 125.3G11 | I + PAR | TAMU |
| 125H6 | IV | CHORI241 |
| 126G2 | II | CHORI241 |
| 129K23 | I multicopy | CHORI241 |
| 131N23 | V | CHORI241 |
| 132K10 | III | CHORI241 |
| 132N15 | II | CHORI241 |
| 134I16 | I multicopy | CHORI241 |
| 137I17 | I + PAR | CHORI241 |
| 139C20 | I multicopy | CHORI241 |
| 140J20 | I multicopy | CHORI241 |
| 140M23 | I multicopy | CHORI241 |
| 142O2 | I multicopy | CHORI241 |
| 144B9 | V+PAR | CHORI241 |
| 147K8 | I multicopy | CHORI241 |
| 148G3 | II | CHORI241 |
| 149H8 | II | CHORI241 |
| 152 E2 | III | CHORI241 |
| 152G20 | I multicopy | CHORI241 |
| 155B8 | II | CHORI241 |
| 155M11 | II | CHORI241 |
| 159 E3 | V | CHORI241 |
| 159F5 | II | CHORI241 |
| 160K10 | I multicopy | CHORI241 |
| 165 E24 | I multicopy | CHORI241 |
| 167N20 | III | CHORI241 |
| 168I4 | II | CHORI241 |
| 168O8 | V | CHORI241 |
| 172 E14 | I + PAR | CHORI241 |
| 172D14 | I + PAR | CHORI241 |
| 172I8 | II | CHORI241 |
| 179K8 | IV | CHORI241 |
| 180P20 | IV | CHORI241 |
| 181B18 | I multicopy | CHORI241 |
| 185M14 | II | CHORI241 |
| 186J13 | I | CHORI241 |
| 188 E20 | II | CHORI241 |
| 190M2 | II | CHORI241 |
| 205D10 | IV | CHORI241 |
| 207P5 | I + PAR | CHORI241 |
| 209K10 | II | CHORI241 |
| 215C6 | III | CHORI241 |
| 244B13 | I + PAR | CHORI241 |
| 264G20 | I + PAR | CHORI241 |
| 269J9 | III | CHORI241 |
| 272B4 | I multicopy | CHORI241 |
| 275P16 | I multicopy | CHORI241 |
| 278M12 | II | CHORI241 |
| 280P20 | I multicopy | CHORI241 |
| 291D19 | II | CHORI241 |
| 318M1 | IV | CHORI241 |
| 331 E10 | IV | CHORI241 |
| 341G20 | II | CHORI241 |
| 377O23 | II | CHORI241 |
| 394K12 | I multicopy | CHORI241 |
| 395L19 | II | CHORI241 |
| 406I22 | I multicopy | CHORI241 |
| 415H8 | I multicopy | CHORI241 |
| 417N24 | II | CHORI241 |
| 418J18 | I + PAR | CHORI241 |
| 422 E23 | II | CHORI241 |
| 437I11 | II | CHORI241 |
| 450C22 | IV | CHORI241 |
| 456J9 | I multicopy | CHORI241 |
| 504H13 | V | CHORI241 |
| 510F11 | I multicopy | CHORI241 |
| ABW | I multicopy | INRA |
| BBW | I multicopy | INRA |
| CBW | I multicopy | INRA |
| DBW | I multicopy | INRA |
| EBW | II | INRA |
| FBW | II | INRA |
| GBW | II | INRA |
| HBW | I multicopy | INRA |
| JBW | I multicopy | INRA |
| LBW | I | INRA |
| MBW | I | INRA |
| OBW | II | INRA |
