# Supplementary material for: A Gene Catalogue of the Euchromatic Male-Specific Region of the Horse Y Chromosome: Comparison with Human and Other Mammals
Source: PLoS One. 2011 Jul 25;6(7):e21374. doi: 10.1371/journal.pone.0021374 (PMC3143126; doi:10.1371/journal.pone.0021374)
Supplement: Table S3 — Primer for 3′ and 5′ RACE PCR (DOC) [file pone.0021374.s005.doc]

**Table S3. Primer for 3’ and 5’ RACE PCR**

| **Marker** | **Primer** | **Sequence 5′ to 3′** |
| --- | --- | --- |
| ETSTY1 | 5′R | TCAGGGCCAAAAACACAAGGTCGTC |
|  | Nested-5′R | AAAACACAAGGTCGTCCGTCCAC |
|  | 3′’F | CTTTCCAGATCTGCTCCGTGTGACT |
|  | Nested-3′F | GATCTGCTCCGTGTGACTGGTGCTA |
| ETSTY2 | 5′R | GGAGGTGTGGTTACTCTCCTTGGGTTGA |
|  | Nested -5′R | TGGTTACTCTCCTTGGGTTGAGTGC |
|  | 3′F | CAGCCCAAAGAAGTAACCGACACG |
|  | Nested -3′F | CAGAAAGCCAAACCACAGCCTCTTC |
| ETSTY3 | 5′R | CCCGGGAAGGCTAACCGGAAACTAT |
|  | Nested -5′R | CTCTGCCAAAGCTGACTGAGGAAAC |
|  | 3′F | ATGTGTAGGGCCAGTTGAACAGCAG |
|  | Nested -3′F | CCAAAGAAAAACCCAGCCTCAGC |
| ETSTY4 | 5′R | GCTGTGGAGGTTATGGTTTGCCCTTG |
|  | Nested -5′R | CGGTTTGGTTAGTTCGTGGAGGTT |
|  | 3′F | AACCGTACCTTCTGCAGCAACCAG |
|  | Nested -3′F | AGGGAGCCTAGCCACAAACTGCAC |
| ETSTY5 | 5′R | TGCTCCAACTTGCCTCTCTTTTGCAG |
|  | Nested -5′R | CTCTGCCTTTACGCATTCCCTCATAC |
|  | 3′F | AAGCTCACGGCATAACGGGCTAGTA |
|  | Nested -3′F | CTGGAGGCGACGAAGTACCTCAGAA |
| ETSTY6 | 5′R | GGGTCCTGGGTCAGTTACCACAGAGG |
|  | Nested -5′R | GCAGATGTAGTCTGGCTTCCTGGAT |
|  | 3′F | ATACAGACGTGCTCCCAGGCACTT |
|  | Nested -3′F | AGGACACTGGTGGCCTTGGTCTCT |
| TSPY | 5′R | TGGCTGACATCTGGGGGTGGTTCA |
|  | Nested -5′R | TGGCTGACATCTGGGGGTGGTTCA |
|  | 3′F | GCAGCCGGAGACGGGACTGAGAGTAGG |
|  | Nested -3′F | GCAGCCGGAGACGGGACTGAGAGTAGG |
| RBMY | 5′R | GGGGGAGAGGCGTATATTGGCTTT |
|  | Nested -5′R | CGATGTGAAAGAGAAGGCCGAAG |
|  | 3′F | CAGAGGGAGTGCTCGTGGTG |
|  | N-3′F | GCTCGTGGTGGTGGCGCACC |
| GeneRacer™ | 5′ F | CGACTGGAGCACGAGGACACTGA |
|  | 5′ Nested F | GGACACTGACATGGACTGAAGGAGTA |
|  | 3′ R | GCTGTCAACGATACGCTACGTAACG |
|  | 3′ Nested R | CGCTACGTAACGGCATGACAGTG |
